# The Collegian of 1666 and the Collegians of 1885; or, What is Recognised Treatment?

**Published:** 1885-09

**Authors:** 


*The Collegian of* 1666 *and the Collegians of* 1885; *or, What is Recognised Treatment?* Pp. 118. London: LEWIS. 1885.

We must read this book to discover its author and his purpose. It is written by Mr. H. O. Thomas, of Liverpool, and it is intended to prove that Sydenham and Mr. Thomas alone among medical men have properly understood and taught the treatment of Intestinal Obstruction by "rest, starvation, and opium." Mr. Thomas seeks to support this proposition by "evidence which shall equal any confirmation required, outside of mathematics," expressed in English that is always vigorous in words, but somewhat weak in grammar, and positively demoralised as to spelling. For the spelling, Mr. Thomas is probably in no way responsible, but it jars on one to meet such words on one page as "laparrotomy," "publically," "antisceptic;" and over the page to meet such a sentence as this—"This operation is the making of an opening in the abdominal wall and stitching to this gap, a presenting portion of bowel and there incisins it." If Mr. Thomas was his own proof-reader on this occasion, we advise him on no consideration to do himself this injustice again.

With all its drawbacks, the book contains some clever and close criticism of the most recent writings on the treatment of Intestinal Obstruction. Mr. Mitchell Banks and Mr. Treves, in particular, he has pursued step by step, throughout a veritable labyrinth of mazy windings, to prove in wholesale and in detail that neither one of them has properly understood or appreciated Sydenham or himself. Mr. Bryant does not escape, and a good many others are made to feel the weight of Mr. Thomas's lash.

With the exception of those whom he has directly influenced, Mr. Thomas tells us that no practitioners have as yet proved themselves competent to treat Intestinal Obstruction. It is impossible to withhold our admiration from a man who makes a statement of this sort. It is almost as if he were preaching a crusade. He evidently believes in himself and his measures, and the very enthusiasm of the man is infectious.

In calm and contemplative moments, the reviewer has had an uncomfortable feeling that he has seen and practised too much of the "rest, opium, and starvation" treatment; but the perusal of Dr. Thomas's work has soothed his conscience, and will very likely induce him to try it again. He is not quite sure how he is to ring the changes on his past practice, unless it be by more rest, more starvation, and more opium; but he means to purchase Mr. Thomas's large work on Intestinal Obstruction, and try and find out.

